# Human plasma plasminogen internalization route in *Plasmodium falciparum*-infected erythrocytes

**DOI:** 10.1186/s12936-020-03377-4

**Published:** 2020-08-26

**Authors:** Sarah El Chamy Maluf, Marcelo Yudi Icimoto, Pollyana Maria Saud Melo, Alexandre Budu, Rita Coimbra, Marcos Leoni Gazarini, Adriana Karaoglanovic Carmona

**Affiliations:** 1 Departamento de Biofísica, Universidade Federal de São Paulo, Rua Pedro de Toledo 669, 7°andar, Vila Clementino, São Paulo, 04039032 Brazil; 2 Centro de Microscopia Eletrônica (CEME), Universidade Federal de São Paulo, Rua Botucatu 862, Vila Clementino, São Paulo, Brazil; 3 Departamento de Biociências, Universidade Federal de São Paulo, Rua Silva Jardim 136, Lab. 329, 3°andar, Vila Mathias, Santos, São Paulo, 11015020 Brazil

**Keywords:** *Plasmodium falciparum*, Protein uptake, Plasminogen, Malaria

## Abstract

**Background:**

The intra-erythrocytic development of the malaria parasite *Plasmodium falciparum* depends on the uptake of a number of essential nutrients from the host cell and blood plasma. It is widely recognized that the parasite imports low molecular weight solutes from the plasma and the consumption of these nutrients by *P. falciparum* has been extensively analysed. However, although it was already shown that the parasite also imports functional proteins from the vertebrate host, the internalization route through the different infected erythrocyte membranes has not yet been elucidated. In order to further understand the uptake mechanism, the study examined the trafficking of human plasminogen from the extracellular medium into *P. falciparum*-infected red blood cells.

**Methods:**

*Plasmodium falciparum* clone 3D7 was cultured in standard HEPES-buffered RPMI 1640 medium supplemented with 0.5% AlbuMAX. Exogenous human plasminogen was added to the *P. falciparum* culture and the uptake of this protein by the parasites was analysed by electron microscopy and Western blotting. Immunoprecipitation and mass spectrometry were performed to investigate possible protein interactions that may assist plasminogen import into infected erythrocytes. The effect of pharmacological inhibitors of different cellular physiological processes in plasminogen uptake was also tested.

**Results:**

It was observed that plasminogen was selectively internalized by *P. falciparum*-infected erythrocytes, with localization in plasma membrane erythrocyte and parasite’s cytosol. The protein was not detected in parasitic food vacuole and haemoglobin-containing vesicles. Furthermore, in erythrocyte cytoplasm, plasminogen was associated with the parasite-derived membranous structures tubovesicular network (TVN) and Maurer’s clefts. Several proteins were identified in immunoprecipitation assay and may be involved in the delivery of plasminogen across the *P. falciparum* multiple compartments.

**Conclusion:**

The findings here reported reveal new features regarding the acquisition of plasma proteins of the host by *P. falciparum*-infected erythrocytes, a mechanism that involves the exomembrane system, which is distinct from the haemoglobin uptake, clarifying a route that may be potentially targeted for inhibition studies.

## Background

In 2018, malaria caused 228 million infections and over 400,000 deaths worldwide, predominantly in children under 5 years of age in Africa. The deaths were primarily due to infection by *Plasmodium falciparum*, the most virulent of the human malaria parasites [1]. All the clinical symptoms of malaria are a consequence of the intra-erythrocytic development of the parasite [2]. In this life cycle stage, it invades red blood cells (RBCs), wherein it resides in a compartment termed the parasitophorous vacuole (PV), where a single parasite, reproducing asexually, is capable of generating 16 to 32 merozoites within 48 h [2, 3].

The rapid growth and replication of parasites require a high nutrient demand, leading to extensive digestion of host cell haemoglobin. However, the RBC content does not provide all the nutrients to sustain parasite growth, as haemoglobin does not contain isoleucine, and several other essential amino acids, such as glutamate, methionine, cysteine, and proline, are under-represented [4–7]. In addition, parasites require purine precursors and pantothenate from the plasma [8, 9]. When isoleucine is withdrawn from the culture medium of intraerythrocytic *P. falciparum*, the parasite slows its metabolism and progresses through its developmental cycle at a reduced rate, entering in a hibernation state. Protein degradation during starvation is important for maintenance of this state [10].

Trafficking pathways in malaria-infected erythrocytes are complex and solutes from the extracellular medium must cross over three membranes, namely, those of RBCs, of the parasitophorous vacuole and of the parasite [5, 9]. While many of these metabolites can be transported across the erythrocyte plasma membrane, others are not or are transported at a rate that is insufficient to sustain rapid parasite growth [7–9, 11, 12]. To facilitate nutrient acquisition from the extracellular environment, *P. falciparum* extensively modifies its host cells and induces the expression of new permeability pathways (NPPs) in the erythrocyte membrane [13, 14]. The NPPs allow faster permeation of a diverse range of low-molecular-mass solutes, including monosaccharides and other polyols, amino acids, nucleosides, and various organic and inorganic ions [13–16].

To perform these modifications, malarial parasites export hundreds of effector proteins into their host cells (the host-targeting ‘secretome’), and this protein trafficking depends on the appearance of membrane systems and compartments in the erythrocyte cytosol, collectively referred as the exomembrane system, which includes the tubovesicular network (TVN), Maurer’s clefts, electron-dense vesicles (∼ 80 nm diameter), and J-dots (protein complex mobile structures) [3, 17]. The tubovesicular network (TVN) is an extension of the parasitophorous vacuole membrane that elongates into the host cell cytosol, while Maurer’s clefts are mobile membranous structures that bud from the PVM in early ring-stage development and become physically tethered to the host cell membrane in the trophozoite stage [17, 18].

Most studies of incorporation and utilization of host factors have focused on low-molecular-weight compounds such as sugars, lipids, vitamins, and ions [19–25]. However, in recent decades, there has been accumulating evidence for the import of functional proteins by malaria-infected erythrocytes. El Tahir et al. [26] described the import from the surrounding milieu of five proteins with molecular weights in the range of 45 to 206 kDa, including albumin, to the parasite within the erythrocyte. Koncarevic and co-workers [27] demonstrated that *P. falciparum* imports from the host erythrocyte into its cytosol the human redox-active protein peroxiredoxin 2 (hPrx-2), which plays a biologically relevant role in the detoxification of hydroperoxides in parasites. More recently, Tougan et al*.* [28] showed that human vitronectin is internalized into *P. falciparum*-infected erythrocytes during the schizont stage and co-localizes on the merozoite surface. The authors observed that vitronectin binding in merozoite-mimic beads prevented their engulfment by THP-1 monocytes functioning in the evasion of the host’s immune response.

Previous studies have reported that the serum proteins kininogen and plasminogen are observed in iRBCs and are intracellularly hydrolysed by malaria parasite proteases, releasing fragments that may modulate host physiology during malarial infections [29, 30]. Plasminogen is a 93 kDa multifunctional glycoprotein that is activated by plasminogen activators to generate plasmin, the major enzyme responsible for fibrin clot degradation in vivo [31]. In addition to fibrinolysis, the plasminogen/plasmin system plays roles in wound healing, cell signalling, extracellular matrix degradation, and inflammatory regulation [31]. Another fragment of plasminogen is angiostatin (38 kDa), a potent endogenous inhibitor of mesenchymal stem cells, endothelial cells and angiogenesis [31].

Despite accumulating evidence about the importation of functional proteins by the parasite, the mechanisms involved in this process have not yet been elucidated, and the route of external medium solute entry into the parasite cytoplasm is poorly investigated. In the present work, different experimental approaches were used to analyse the internalization of human plasma plasminogen in *P. falciparum*-infected erythrocytes, aiming to provide new elements for the understanding of large solute trafficking through different membranes, clarifying the route that may be unique and potentially targeted for inhibition.

## Methods

### *Plasmodium falciparum* culture

*Plasmodium falciparum* clone 3D7 was cultured following the Trager and Jensen [32] method using standard HEPES-buffered RPMI 1640 medium (Gibco Life Technologies) supplemented with 0.5% AlbuMAX II (Life Technologies, UK). Parasite synchronization at ring stages was performed with 5% (w/v) D-sorbitol solution as described by Lambros and Vanderberg [33], and parasitaemia was determined by standard Giemsa staining and blood smear microscopy.

### Fractionation of uninfected and infected erythrocytes

Purification of uninfected erythrocyte membranes was performed as described by Low et al. [34]. Briefly, the red blood cells (RBCs) were resuspended in three volumes of 0.9% w/v NaCl in 5 mM phosphate buffer at pH 8.0 and centrifuged at 500 g for 10 min (repeated 3 times). RBCs were lysed with cold 5 mM phosphate buffer, pH 8.0, containing 1 mM EDTA and a protease inhibitor cocktail (10 µM E-64, 1 mM PMSF, 1 µM pepstatin and 10 mM *ortho*-phenathrolin). The lysate was then centrifuged at 100,000*g* at 4 °C for 30 min in a Beckman L5-50B ultracentrifuge with a Ti 75 rotor. The supernatant (RBC cytosol fraction) was collected, and the membrane pellet was resuspended separately in the same buffer (RBC plasma membrane fraction).

The preparation of infected erythrocyte fractions was performed according to Hsiao and co-workers [35], with some modifications. Packed trophozoite-infected erythrocytes were resuspended in PBS (137 mM NaCl, 2.7 mM KCl, 4.3 mM Na_2_HPO_4_, 1.4 mM NaH_2_PO_4_) in the presence of the protease inhibitor cocktail cited above and lysed with 0.1% (w/v) saponin at room temperature for 10 min. The lysate was centrifuged at 16,000*g* for 1 min at room temperature. Isolated parasites were pelleted in the bottom, and the fragments of the erythrocyte membrane formed a visible band above the parasite pellet. The supernatant containing the iRBC cytosol fraction was collected in a new tube. The erythrocyte membrane layer was collected and placed in PBS with a protease inhibitor cocktail and centrifuged at 100,000*g* at 4 °C for 30 min. The resulting pellet was resuspended in the same buffer (iRBC plasma membrane fraction). Parasite pellets were disrupted by hypotonic lysis (1 mM HEPES, pH 7.2) on ice for 1 h in the presence of the protease inhibitor cocktail. To remove cell debris and plasma membranes, the lysate was centrifuged at 100,000*g* at 4 °C for 30 min, and the supernatant (parasite cytosol fraction) was collected. All fractions were stored at 80 °C until subjected to SDS-PAGE and Western blotting.

### Treatment of parasites with pharmacological inhibitors of cell physiology processes

To further understand the mechanisms involved in plasminogen trafficking into infected RBCs, it was investigated the effect of inhibitors of different cellular processes on their uptake. Trophozoite-stage parasites at ~ 30% parasitaemia were incubated in PBS for 1 h, at 37 °C, with one the following compounds: 1 µM antimycin (Sigma-Aldrich, USA), 10 mM sodium azide, (Sigma-Aldrich, USA), 20 µM brefeldin (Sigma-Aldrich, USA), 1.3 µM cycloheximide (Sigma-Aldrich, USA), 10 µM FCCP + 10 mM 2-deoxy-d-glucose (Sigma-Aldrich, USA) or 50 µM genistein (Sigma-Aldrich, USA). After the treatment, samples were washed three times (300*g*, 2 min) with PBS and incubated with 4 µg/mL plasminogen (R&D Systems, USA) for 1 h at 37 °C. After incubation and washings, the parasite cytosol was purified as described in the fractionation section, and the effect of each treatment on plasminogen internalization was analysed by Western blotting to detect the presence of the human protein in the parasite.

### Plasminogen western blot analysis

Aliquots from each obtained cell fraction were resuspended in SDS-containing sample buffer and resolved on 10–12% Bis–Tris gels. The separated proteins were transferred onto polyvinylidene fluoride membranes and incubated in blocking buffer [PBS containing 0.05% (v/v) Tween 20 (PBS-T) with 5% (w/v) BSA] for 1 h. The primary antibodies were incubated overnight at 4 °C: anti-angiostatin (1:1000) (R&D Systems, USA), anti-Pfaldolase (1:1000) (Santa Cruz, USA), and anti-band3 (1:5000) (Sigma-Aldrich, USA) diluted in blocking buffer. After three washes with PBS-T, the membranes were incubated for 1 h in blocking buffer containing the secondary antibody anti-mouse IgG-HRP (1:1000). Membranes were washed with PBS-T and incubated with SuperSignal West Pico Chemiluminescent Substrate and imaged using the Odyssey® Fc Imaging System.

### Identification of plasminogen-associated proteins by immunoprecipitation and mass spectrometry analysis

Flasks of asynchronous culture parasite (six negative control and six with plasminogen) with 30% parasitaemia collected from 3 consecutive days, were incubated with 4 µg/mL plasminogen (R&D Systems, USA) for 1 h at 37 °C. The control consisted of a protein-negative culture without the addition of human plasminogen. After three washes with PBS buffer to remove any plasminogen that hasn't been taken up, parasites were isolated with saponin as described above. After three washes with PBS (16,000*g*, 1 min), parasite pellets were suspended in Hanks’ Balanced Salt Solution (25 mM HEPES, 121 mM NaCl, 5 mM NaHCO_3_, 4.7 mM KCl, 1.2 mM KH_2_PO_4_, 1.2 mM MgSO_4_, 10 mM D-glucose, pH 7.4), and the chemical crosslinking of intracellular proteins was performed with 2 mM of the cell-permeable crosslinker DSS (Thermo Fisher Scientific Inc., USA) for 30 min at room temperature. Reactions were stopped by quenching DSS with 2 mM Tris–HCl (pH 7.4) for 15 min at room temperature.

Parasites were lysed with 1% Triton X-100 in PBS containing protease inhibitor cocktail for 30 min on ice and centrifuged at 16,000*g* for 10 min at 4 °C. The supernatant was used for IP analyses. The lysates were incubated overnight at 4 °C on a rotary mixer with Dynabeads® M-270 epoxy conjugated with 20 µg of anti-angiostatin antibody. After incubation, the beads were washed three times with 1% Triton X-100 in PBS, and bound proteins were eluted from the beads using 100 mM glycine, pH 2.6. The released proteins were separated by SDS-PAGE and run for only 0.5 cm. The regions containing the bands of interest were cut from the gel, and the digestion and identification of the proteins by mass spectrometry were performed by the Taplin Biological Mass Spectrometry Facility at Harvard Medical School (Boston, USA).

### Immunogold electron microscopy (IEM)

Asynchronous parasite cultures were incubated with 4 µg/mL plasminogen (R&D Systems, USA) for 1 h at 37 °C. For immunoelectron microscopy of *P. falciparum*-infected erythrocytes, the previously described pre-embedding silver enhancement immunogold method was used with modifications [36]. The parasitized erythrocytes were fixed in 4% paraformaldehyde and 0.0075% glutaraldehyde dissolved in 0.1 M sodium phosphate buffer (pH 7.4) for 2 h and then washed three times (300*g*, 2 min) with PBS. Then, the cells were permeabilized in liquid nitrogen and incubated in a blocking buffer containing 0.005% saponin, 10% goat serum, 0.1% cold water fish gelatine, and 10% bovine serum albumin for 30 min and reacted overnight with mouse anti-angiostatin (1:250) in blocking buffer at 4 °C. Next, the cells were washed (300*g*, 2 min) in PBS containing 0.005% saponin and incubated with goat anti-mouse IgG conjugated with colloidal gold (1.4-nm diameter, Nanogold, Nanoprobes) (1:10) in blocking buffer for 2 h at room temperature. Cells were washed five times with PBS containing 0.005% saponin for 10 min, washed with PBS for 5 min, and fixed with 1% glutaraldehyde for 10 min. After washing, the gold particles were intensified using a silver enhancement kit (HQ silver, Nanoprobes) for 6 min at 20 °C in the dark. Cells were then centrifuged at 10,000*g* for 2 min and the pellets containing the *P. falciparum*-infected erythrocytes were incorporated to Agar 5% for further processing in epoxi resin. Next, the blocks were suspended and dehydrated with a graded ethanol, incubated in propylene oxide and embedded in Epon resin. Ulltrathin sections (70 nm) were doubly stained with uranyl acetate and lead citrate and observed at transmission electron microscopy at 80 kV (JEOL, EX II 1200, Japan). Images were acquired with a camera connected to microscope (GATAN Orius, USA).

## Results

### Plasminogen uptake in the cytosol of *Plasmodium falciparum*

**Fig. 1:**
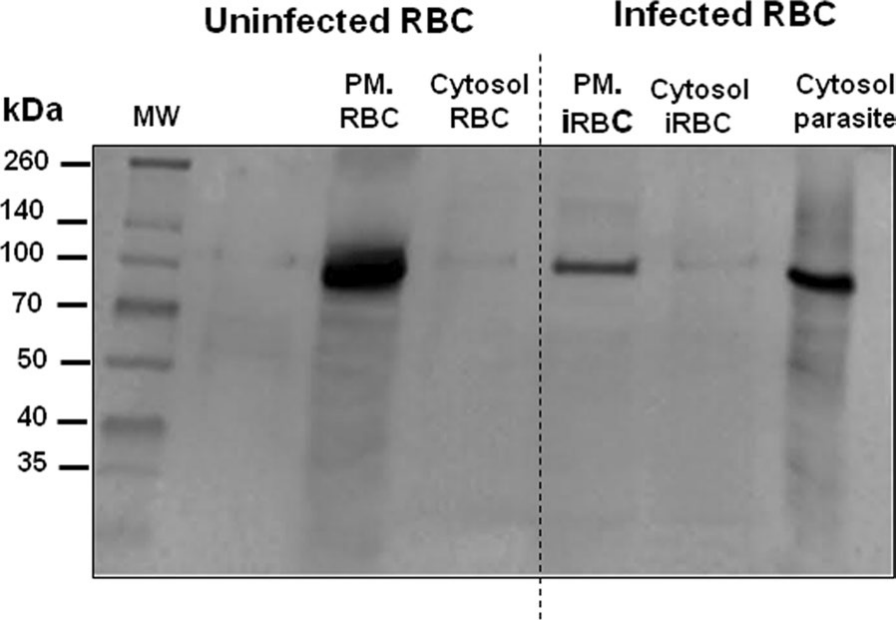
Detection of human plasminogen in cellular fractions from uninfected and infected erythrocytes by Western blotting. The uninfected erythrocytes were lysed by hypotonic solution, and the plasma membrane fraction was separated from the cytosol by centrifugation. Trophozoite-infected erythrocytes were lysed with saponin, and the supernatant containing the cytosol and plasma membrane of infected erythrocytes was separated by 100,000*g* ultracentrifugation. The parasite pellet was lysed by hypotonic solution and subjected to ultracentrifugation to eliminate membrane fractions and obtain only the parasite cytoplasm fraction

Cellular fractionation of uninfected and infected RBCs was performed to investigate plasminogen localization in the different cell compartments, and the sub-cellular presence of the human protein was demonstrated by Western blotting analysis. Uninfected erythrocytes possess plasminogen associated with the plasma membrane; however, the protein had not been detected in significant amounts in the RBC cytoplasm (Fig. 1). In infected erythrocytes, part of the plasminogen added to the culture was located in the cytosol of *P. falciparum,* and part remained associated with the iRBC plasma membrane. Interestingly, plasminogen was not detected in the erythrocyte cytosol (Fig. 1). These results suggested that human plasminogen from extracellular medium can access the parasite cytosol inside erythrocytes.

### Human plasminogen distribution in *Plasmodium falciparum*-infected erythrocytes detected by immunogold electron microscopy (IEM)

**Fig. 2:**
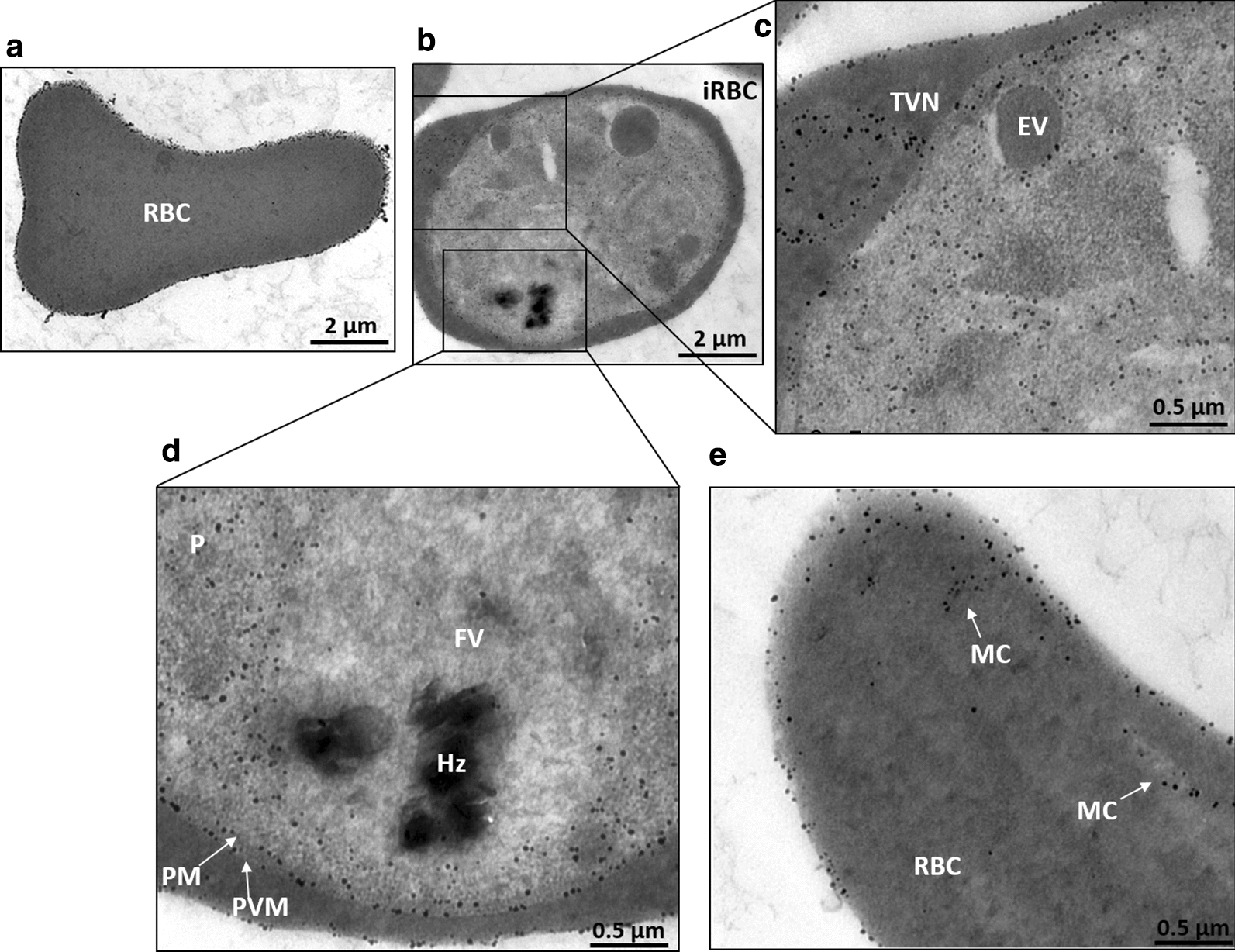
Transmission electron microscopy micrographs of *Plasmodium falciparum*-infected erythrocytes with immunogold-labelled plasminogen. Uninfected and infected erythrocytes were analysed by immunoelectron microscopy (immunogold and silver enhancement method) with an antibody against angiostatin. **a** The immunogold particles (black dots) are associated with uninfected erythrocyte plasma membrane; **b** iRBCs with parasites at the trophozoite stage; **c**, **d** magnified images of the areas indicated in (**b**). Images show the presence of human plasminogen in the parasite cytosol (not significant inside organelles) and its presence in the three membranes (RBC, PVM and PM). Food vacuoles (FVs) containing residual hemozoin (HZ) and endocytic vesicles (EVs) of RBC haemoglobin are not labelled. The analyses also show the human protein in and attached to the tubovesicular network (TVN) and **e** Maurer’s clefts (MC)

The IEM micrographs are able to define the subcellular localization of plasminogen. In uninfected RBCs, the gold particles were founded consistently associated with the plasma membrane surface, while labelling was not detected in the cell cytoplasm (Fig. 2a). In infected RBCs, electron micrographs showed intense plasminogen staining located in the parasite cytoplasm and in the iRBC plasma membrane. In addition, the protein was also observed in the parasitophorous vacuolar space. Furthermore, the ultrastructural analysis revealed another interesting feature of the distribution pattern: when plasminogen is present in the iRBC cytoplasm, it is associated with the exomembrane system lumen, such as the tubovesicular network (TVN) and Maurer’s clefts (MCs) (Figs. 2b, c, e), revealing the protein trafficking route. The observed plasminogen staining pattern in these structures is comparable to other published IEMs [18]. Interestingly, plasminogen signals were not detected on the FV containing residual hemozoin (Fig. 2d) or in endocytic vesicles containing haemoglobin (Fig. 2c).

### Parasite proteins that interact with plasminogen

Immunoprecipitation followed by mass spectrometry analysis were used to investigate global plasminogen interactions that may assist this protein import into infected erythrocytes. Among all proteins identified (Additional file 1: Table S1), *P. falciparum* proteins that were unique (unique peptides negative = 0) to, or ≥ threefold enriched (unique peptides positive ≥ 3) in plasminogen immunoprecipitation were selected compared to a plasminogen negative control (Table 1). Furthermore, the enriched proteins with Relative Values above 3 were selected. With this analysis, it was possible to evaluate relevant interacting partners that may be involved in plasminogen transport in *P. falciparum*-infected erythrocytes, as *P. falciparum* Maurer’s cleft 2 transmembrane (PF3D7_1039700), Pf113 (PF3D7_1420700), a recently described PTEX-associated protein [37], vacuolar protein sorting-associated proteins (PF3D7_1250300, PF3D7_1110500). The study also identified the cyto-adherence linked asexual protein 3.1 (PF3D7_0302500) and the cyto-adherence linked asexual protein 3.2 (PF3D7_0302200), that together with RhopH2 and RhopH3 contribute to the nutrient uptake via PSAC (the plasmodial surface anion channel).

**Table 1:** Mass spectrometry analysis of *Plasmodium falciparum* proteins unique to, or ≥ threefold enriched in plasminogen immunoprecipitation compared to a plasminogen negative control

PlasmoDB gene ID	Annotated protein name	Unique peptides negative	Unique peptides positive	Relative values*
PF3D7_0608700	T-complex protein 1 subunit zeta	1	5	24.5
PF3D7_1145400	Dynamin-like protein	15	26	15.5
PF3D7_1015600	Heat shock protein 60	3	11	6.7
PF3D7_0708800	Heat shock protein 110	1	5	4.7
PF3D7_1232100	60 kDa chaperonin	3	9	3.9
PF3D7_0905400	High molecular weight rhoptry protein 3	4	8	3.5
PF3D7_1132200	T-complex protein 1 subunit alpha	3	6	3.5
PF3D7_0929400	High molecular weight rhoptry protein 2	3	15	3.5
PF3D7_0727400	Proteasome subunit alpha type-5, putative	4	5	3.3
PF3D7_1434300	Hsp70/Hsp90 organizing protein	1	3	3.2
PF3D7_0817700	Rhoptry neck protein 5	0	6	1.0
PF3D7_1116000	Rhoptry neck protein 4	0	4	1.0
PF3D7_1320600	Ras-related protein Rab-11A	0	3	1.0
PF3D7_0807500	Proteasome subunit alpha type-6, putative	0	3	1.0
PF3D7_0302200	Cytoadherence linked asexual protein 3.2	0	3	1.0
PF3D7_0214000	T-complex protein 1 subunit theta	0	2	1.0
PF3D7_1250300	Vacuolar protein sorting-associated protein 26, putative	0	2	1.0
PF3D7_1110500	Vacuolar protein sorting-associated protein 35, putative	0	2	1.0
PF3D7_1452000	Rhoptry neck protein 2	0	2	1.0
PF3D7_1039700	PfMC-2TM Maurer's cleft two transmembrane protein	0	2	1.0
PF3D7_1429800	Coatomer subunit beta, putative	0	2	1.0
PF3D7_1134800	Coatomer subunit delta	0	2	1.0
PF3D7_1252100	Rhoptry neck protein 3	0	2	1.0
PF3D7_1118200	Heat shock protein 90, putative	0	2	1.0
PF3D7_1361800	Glideosome-associated connector	0	2	1.0
PF3D7_1420700	Surface protein P113	0	1	1.0
PF3D7_0302500	Cytoadherence linked asexual protein 3.1	0	1	1.0
PF3D7_1211400	Heat shock protein DNAJ homologue Pfj4	0	1	1.0
PF3D7_1231100	Ras-related protein Rab-2	0	1	1.0

Some regulators of vesicular trafficking were also identified, including Ras-related protein Rab-11A (PF3D7_1320600), Ras-related protein Rab-2 (PF3D7_1231100), Coatomer subunits (PF3D7_1429800, PF3D7_1134800) and Dynamin-like protein (PF3D7_1145400). Furthermore, several proteins that belong to the parasite's chaperonin system were detected, among them, the T-complex protein 1 sub-units (PF3D7_0608700, PF3D7_1132200), Heat shock protein 60 (PF3D7_1015600), Heat shock protein 110 (PF3D7_0708800), Heat shock protein 90 (PF3D7_1118200), 60 kDa chaperonin (PF3D7_1232100), Hsp70/Hsp90 organizing protein (PF3D7_1434300), Heat shock protein DNAJ homologue Pfj4 (PF3D7_1211400). It should be mentioned other binding partners identified that are involved in erythrocyte invasion, as Rhoptry neck proteins (PF3D7_0905400, PF3D7_0817700, PF3D7_1116000, PF3D7_1452000) and Glideosome-associated connector (PF3D7_1361800). Regarding protein degradation, some sub-units of the parasite’s Proteasome complex (PF3D7_0727400, PF3D7_0807500) were also identified.

Although further combined studies are necessary to validate these plasminogen interactions, the results presented here may contribute to clarifying a possible important role of these proteins in plasminogen transport in *P. falciparum*-infected erythrocytes.

### Treatment of parasites with pharmacological inhibitors of cell physiological processes did not affect plasminogen internalization

**Fig. 3:**
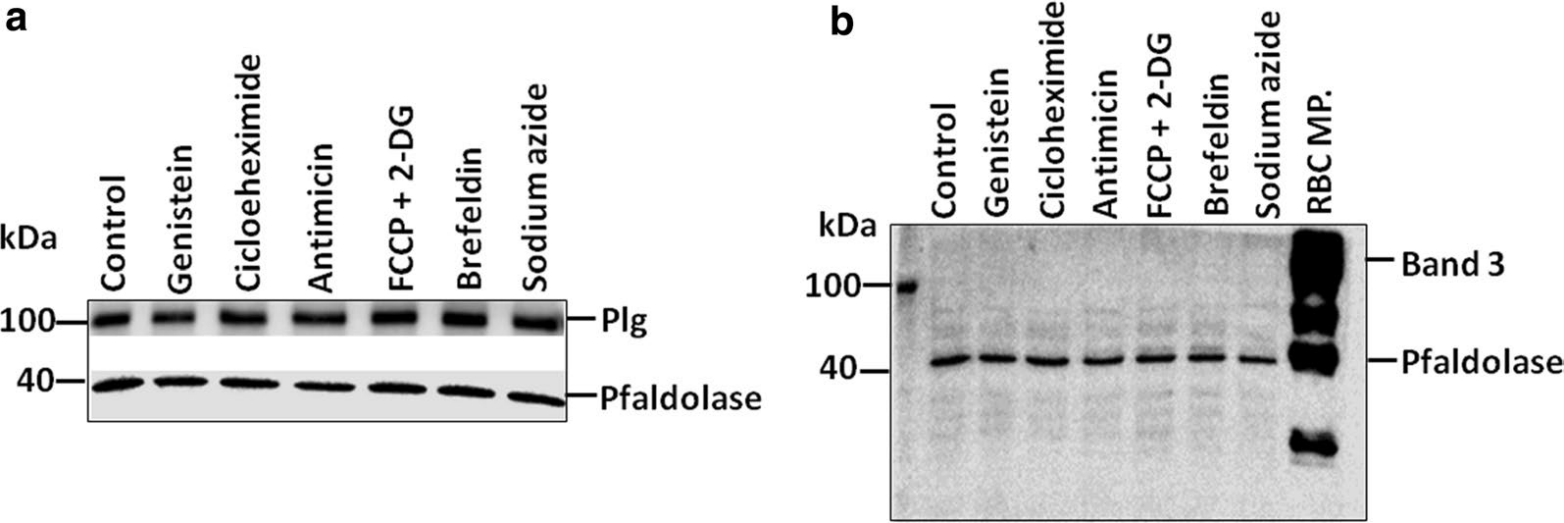
Effect of inhibitors of different cellular processes on plasminogen internalization in infected red blood cells. The effect of each treatment on plasminogen (Plg) import was analysed by Western blotting using anti-angiostatin to detect plasminogen (100 kDa) in the parasite cytosol. **a** Uptake of plasminogen by untreated trophozoite-infected erythrocytes (lane 1) and after 50 µM genistein (lane 2), 1.3 µM cycloheximide (lane 3), 1 µM antimycin (lane 4), 10 µM FCCP + 2-DG (lane 5), 20 µM brefeldin (lane 6) and 10 mM sodium azide treatments (lane 7). **b** The soluble parasite protein Pfaldolase (40 kDa) was used as a loading control. *Plg* plasminogen

The effect of several pharmacological agents that may modulate different cellular processes on the import of plasminogen was investigated. As shown in Fig. 3a, the treatment of infected erythrocytes with antimycin (oxidative phosphorylation inhibitor [38, 39]), sodium azide (oxidative phosphorylation inhibitor [26]), brefeldin (protein transport inhibitor [26, 40]), cycloheximide (protein synthesis inhibitor [41]), FCCP (uncoupler of oxidative phosphorylation in mitochondria [38]), 2-deoxy-d-glucose (glycolysis inhibitor [38]) and genistein (protein tyrosine kinase inhibitor [42]), did not affect the uptake of plasminogen by the parasite. The soluble parasite protein Pfaldolase was used as a loading control. The same samples used were probed with anti-band3, an integral erythrocyte membrane protein, to confirm that samples constituted only the parasite cytosol fraction and were not contaminated with host cell membrane (Fig. 3b).

## Discussion

During the asexual erythrocytic stage of their life cycle, the malaria parasite *P. falciparum* grows and propagates within the RBCs of their vertebrate host [24]. In the course of parasite growth, they extensively modify the erythrocytes by expression and export of hundreds of effector proteins into host cells, which facilitate the acquisition of nutrients from the extracellular environment and the evasion of host immune responses [17]. Studies of protein export mechanisms led to identification of an export motif and the *Plasmodium* translocon of exported proteins (PTEX), a gateway for parasite proteins to traverse the PVM and access the erythrocyte [43]. In contrast, the understanding of the mechanisms of functional protein uptake is at the very beginning stages and is still poorly understood. Pouvelle and co-workers [44] showed that intra-erythrocytic *P. falciparum* can endocytose dextran, protein A and IgG2 antibodies and that these molecules do not cross the erythrocyte or parasitophorous vacuole membranes but rather gain direct access from the external medium to the parasite through a duct, named parasitophorous duct [44]. Other authors have obtained different results, suggesting that the manifestation of the duct might be due to experimental artefacts [45–48]. However, the dependence of serum on intra-erythrocytic growth and development of the parasite, in addition to trafficking of large-molecular-weight compounds, suggests that macromolecular import does take place in this parasite [26–28].

In agreement with previous report [30], here it was demonstrated that human plasminogen is taken up from the external medium into the cytosol of *P. falciparum* (Fig. 1) and the mechanism of the import was analysed. The immunoelectron microscopy micrographs revealed that plasminogen is associated with the tubovesicular network and Maurer’s clefts (Fig. 2c, e). Furthermore, by immunoprecipitation, it was identified the interaction between plasminogen and the protein PfMC-2TM (Table 1). Some studies have revealed that members of the PfMC-2TM family exhibit characteristics of integral membrane proteins, and their location occur in MC, erythrocyte membranes and PV/PVM space [49]. The role of PfMC-2TM proteins in parasite biology is not clear; however, trans-membrane regions of these and other 2TM superfamily members were proposed to provide an anchorage surface to exposed proteins or in the formation of solute ion channels [50]. These data strongly suggest that the exomembrane system, in addition to the previously characterized export pathways to the RBC surface, are involved in protein import mechanisms. The immuno-EM images also showed that plasminogen is located in the PVM/PV/PM space (Fig. 2c, d), parasite’s cytosol, but no significant labelling was seen in the parasitic food vacuole (FV) and haemoglobin-containing vesicle. *P. falciparum* digests host cell haemoglobin, to support parasite growth and asexual replication during the intra-erythrocytic stage.

The internalization of haemoglobin occurs through an unusual structure, the cytostome, an invagination of the parasitophorous vacuole membrane and the parasite membrane [51, 52]. Haemoglobin is then transported through the parasite cytoplasm via vesicular transport and is directed to the food vacuole (FV) [51, 52]. The data here presented suggest that the internalization of plasminogen essentially differs from haemoglobin, as the parasite does not take up plasminogen to degrade it in the FV. As reported by Melo et al. [30], after 30 min of incubation with isolated parasites, exogenous plasminogen is processed on the Leu470-Pro471 peptide bond, with the formation of angiostatin-like fragments recognized by the antibody Human Angiostatin Kringle 1–3. In addition, the authors demonstrated that the generated products are capable to inhibit angiogenesis and to stimulate calcium response in endothelial cells in vitro suggesting that plasminogen uptake and the peptides released may play an important role in the modulation of host local physiology during malarial infections. In this scenario, in a study recently published by Tougan et al. [53] a total of 48 serum proteins were identified in *P. falciparum* lysate, among them, protein S, prothrombin, vitronectin, albumin, plasminogen and kininogen. These proteins were taken up by the parasites in the presence of Ca^2+^, being prothrombin and serum albumin considerably more internalized in iRBCs than the others proteins. The study also showed that thrombin activated by parasite-derived proteases promotes coagulation, providing further insights into the role of thrombin in malaria pathogenicity [53].

The pharmacological inhibitors of different cellular physiological processes did not interfere in plasminogen uptake (Fig. 3). However, the possibility that vesicular transport also contributes to plasminogen traffic in parasite cannot be excluded, since Dynamin-like protein and some regulators of vesicular trafficking, including Ras-related protein Rab-11A, Ras-related protein Rab-2 and Coatomer subunits were immunoprecipitated from Plasminogen (Table 1). These results can serve as a solid starting point for testing other compounds, which different targets, as for example, Cytochalasin D, Dynasore, Furosemide and Jasplakinolide. This approach is used in nutrient transport studies and can help clarifying requirements for protein traffic in infected erythrocytes [26, 51–53]. El Tahir et al. [26] demonstrated a significant decrease in the uptake of biotin labelled HSA (Human Serum Albumin) and recombinant PfHRP-2 (histidine rich protein-2) by *P. falciparum* after sodium azide treatment, suggesting that the import of proteins into the parasites is dependent on ATP. Tougan et al*.* [53] revealed that the uptake of serum proteins was detected in PV lysate treated with Cytochalasin D and Mycalolide B, however, was lower or not detected, in lysate treated with Jasplakinolide.

The immunoprecipitation data here reported indicated the association of plasminogen with other proteins, which may be involved in its traffic from the extracellular medium to the parasite, among them, chaperones such as Heat shocks proteins, T-complex protein 1 subunits (TRiC) and Pfj4 (a type of HSP40) (Table 1). There was also identified Pf113, a PTEX-associated protein [37]. However, further studies are necessary to validate these interactions and evaluate the role of these proteins in plasminogen traffic.

## Conclusion

**Fig. 4:**
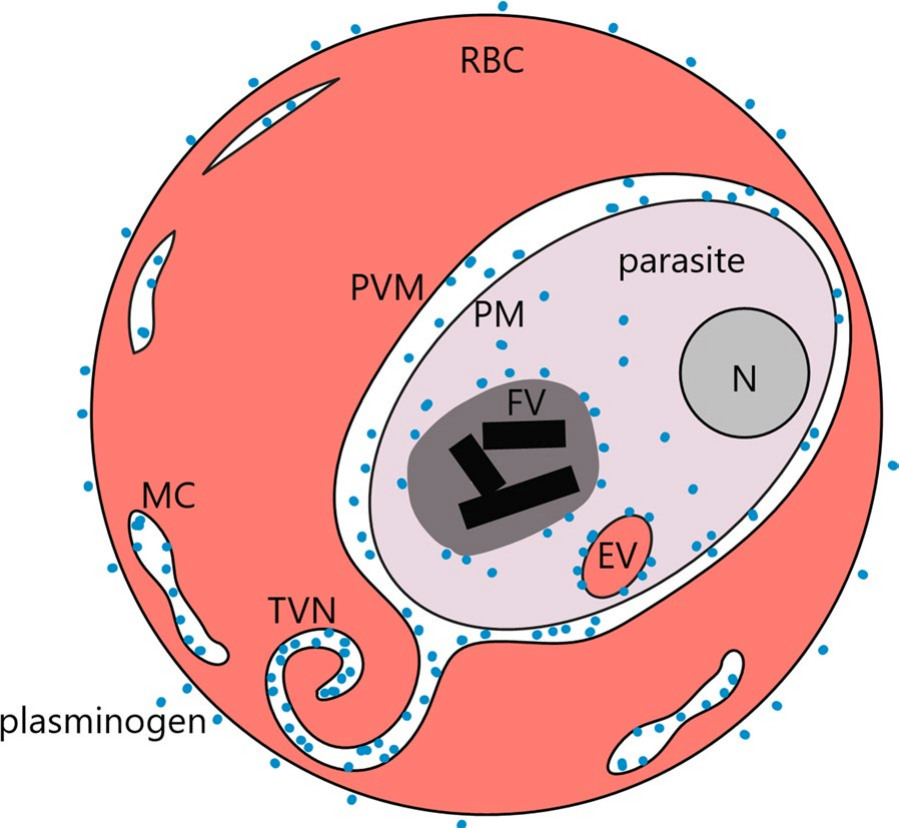
Model proposed for human plasminogen internalization in *Plasmodium falciparum*-infected erythrocytes. A possible route for the transport of plasminogen is through the exomembrane system in cytosolic erythrocytes (MCs, TVNs). Once in the parasitophorous vacuole, plasminogen is transported by a different pathway, with the passage through the PM, from that used for haemoglobin uptake (cytostome-derived endocytic vesicles—EV). Inside the parasite, plasminogen is located in its cytosol. *TVN* tubovesicular Network, *MC* Maurer’s Clefts, *PVM* parasitophorous vacuole membrane, *PM* parasite membrane, *FV* food vacuole, *N* nucleus

Taken together, the results here presented allow us to propose a putative model of a human large protein, as plasminogen, trafficking from the extracellular medium to the parasite cytosol in *P. falciparum-*infected erythrocytes (Fig. 4). The human protein traffics through membranous structures, as tubovesicular network (TVN) and Maurer’s clefts, in a route distinct from the haemoglobin uptake. Understanding these interactions may lead to novel therapeutic approaches based on impairment of functional protein import from the extracellular milieu to *Plasmodium*.

## Supplementary information

**Additional file 1: Table S1.** List of all proteins detected in immunoprecipitation followed by mass spectrometry analyses, with number of peptides (unique and total) and the sum intensity of any given protein in negative and positive plasminogen samples.

## Data Availability

All the data generated during the study are included in this published article and in the additional file.

## Abbreviations

CytD: Cytochalasin D
2-DG: 2-Deoxy-d-glucose
EV: Endocytic vesicles
FCCP: Carbonyl cyanide-4-(trifluoromethoxy) phenylhydrazone
FV: Food vacuole
Hz: Hemozoin
IEM: Immunogold electron microscopy
iRBC: Infected red blood cell
MC: Maurer’s clefts
NPP: New permeability pathway
PBS: Phosphate buffered saline
PfMC-2TM: *P. falciparum* Maurer’s cleft 2 transmembrane
Plg: Plasminogen
PMSF: Phenylmethylsulfonyl fluoride
PM: Plasma membrane
PV: Parasitophorous vacuole
PVM: Parasitophorous vacuole membrane
PTEX: *Plasmodium* translocon of exported proteins
RBC: Red blood cell
RPMI: Roswell Park Memorial Institute 1640
TRiC: t-Complex protein 1 (TCP-1) ring complex
TVN: Tubovesicular network
